# Plasma serotonin precursors and metabolites as diagnostic and therapeutic biomarkers for osteoporosis in postmenopausal women

**DOI:** 10.17305/bb.2025.11513

**Published:** 2025-06-11

**Authors:** Peiying Li, Shuyi Wu, Wenshan Chen, Minjuan Liu

**Affiliations:** 1 Department of Gynecology, The Tenth Affiliated Hospital, Southern Medical University (Dongguan People’s Hospital), Dongguan, Guangdong, China

**Keywords:** Postmenopausal women, plasma, 5-hydroxytryptophan, 5-HTP, 5-hydroxytryptamine, 5-HT, 5-hydroxyindoleacetic acid, 5-HIAA, osteoporosis, diagnosis, target

## Abstract

This study aims to evaluate the diagnostic and therapeutic potential of plasma 5-hydroxytryptamine (5-HT) precursors and metabolites in postmenopausal osteoporosis (PMOP). A total of 287 consecutive postmenopausal women were retrospectively enrolled. Data including age, body mass index (BMI), serum calcium, serum phosphorus, menopausal duration, and bone mineral density (BMD) of the lumbar spine and femoral neck, as well as serum and plasma samples were collected. Based on BMD measurements, participants were categorized into normal, osteopenia, and osteoporosis (OP) groups. Serum β-C-terminal telopeptide of type I collagen and procollagen type I N-propeptide, along with plasma levels of 5-HT precursors and metabolites, were measured using Enzyme-linked immunosorbent assay. Receiver operating characteristic curve analysis, multivariate analysis, and Kaplan–Meier curves were employed to assess the predictive value of 5-HT precursors and metabolites in PMOP and to evaluate the association between their expression levels and PMOP risk. Plasma levels of 5-hydroxytryptophan, 5-HT, and 5-hydroxyindoleacetic acid were elevated in PMOP patients and showed correlations with bone turnover markers and BMD. These biomarkers were identified as independent risk factors for PMOP. Combined analysis of the three biomarkers demonstrated greater predictive value than individual markers. Elevated levels were particularly pronounced in women with ≥12 years since menopause (YSM), and were associated with a higher risk of developing PMOP. In summary, 5-HT precursors and metabolites are significantly associated with bone turnover and BMD in postmenopausal women. They serve as independent risk factors and show strong predictive value for PMOP, suggesting their potential as plasma biomarkers for diagnosis and treatment. Furthermore, their relationship with YSM highlights their promise as therapeutic targets to delay the onset of OP in postmenopausal women.

## Introduction

Osteoporosis (OP) is a metabolic bone disorder characterized by reduced bone strength and an increased risk of fracture, particularly in postmenopausal women [1]. Factors such as hormonal changes, aging, genetic susceptibility, and the immune microenvironment play critical roles in the development of postmenopausal OP (PMOP), with estrogen deficiency long recognized as the primary cause [2–4]. Fragility fractures—especially hip fractures—are the most common and severe complications of PMOP, resulting in significant pain and substantial economic burden for patients [5]. As the global population continues to age, the incidence of PMOP has risen markedly [6]. Therefore, identifying novel therapeutic targets is crucial for developing more effective treatment strategies. 5-hydroxytryptamine (5-HT), a key monoamine neurotransmitter, can be classified into peripheral and central 5-HT based on its site of production [7]. In recent years, accumulating evidence has shown that peripheral 5-HT negatively affects bone formation by inhibiting osteogenesis and promoting bone resorption [8–10]. Earlier studies primarily focused on the role of peripheral 5-HT in respiratory function, vascular tone, gastrointestinal inflammation, and hemostasis until a breakthrough emerged in the study of OP-pseudoglioma syndrome (OPPG) [11]. OPPG, a condition characterized by blindness and OP, is caused by mutations in the low-density lipoprotein receptor-related protein 5 (LRP5) gene [12]. Conversely, gain-of-function mutations in LRP5 lead to increased bone mass [13]. These opposing skeletal phenotypes underscore the critical role of LRP5 in bone metabolism regulation [14]. Animal studies have demonstrated that LRP5 loss-of-function mutations result in OP through elevated peripheral 5-HT levels. In mice lacking both alleles of LRP5, tryptophan hydroxylase 1 expression in intestinal tissue is upregulated, leading to increased peripheral 5-HT levels. This, in turn, is associated with reduced bone mineral density (BMD), disrupted bone microarchitecture, and decreased proliferation and differentiation of primary osteoblasts [15]. These findings suggest that peripheral 5-HT may act directly on its receptors in bone tissue to inhibit osteogenesis and promote bone resorption. 5-HT signaling exerts dual effects on osteoblasts: centrally, brain-derived 5-HT functions as a neurotransmitter that stimulates bone formation and inhibits bone resorption; peripherally, 5-HT acts hormonally to enhance bone remodeling while suppressing bone formation [16]. In particular, peripheral 5-HT inhibits osteoblast proliferation in mice via activation of the 5-HT receptor 1B and the transcription factor cyclic adenosine monophosphate response element-binding protein (CREB) [17]. 5-hydroxytryptophan (5-HTP), the immediate precursor of 5-HT, is synthesized from the essential amino acid L-tryptophan by tryptophan hydroxylase [18]. 5-HTP is widely used as a dietary supplement to increase 5-HT levels [19]. Meanwhile, 5-hydroxyindoleacetic acid (5-HIAA), the primary metabolite of 5-HT [20], has been found to negatively correlate with femur stiffness in rats with chronic kidney disease [21]. However, no studies to date have investigated the roles of 5-HT precursors and metabolites in PMOP.This study included postmenopausal women with normal bone mass, osteopenia, and OP, aiming to evaluate plasma levels of 5-HT precursors and metabolites across these groups. Additionally, we examined their associations with bone turnover markers and assessed their potential diagnostic value in PMOP. 

## Materials and methods

### Sample size estimation

The sample size estimation was performed using G*Power 3.0.10 software (Düsseldorf University, Düsseldorf, Germany). The following parameters were used: power ═ 0.80, α ═ 0.05, effect size ═ 0.25, and number of groups ═ 3. *F*-tests (analysis of variance [ANOVA]: fixed effects, omnibus, one-way) were used to calculate the sample size. The results indicated that the minimum required sample size was 159 (Figure S1).

### Study subjects

This single-center, retrospective cohort study included 329 consecutive PM women admitted to the Tenth Affiliated Hospital of Southern Medical University (Dongguan People’s Hospital) between June 2020 and April 2024. After applying the inclusion and exclusion criteria, 287 PM women were ultimately enrolled. Based on BMD measurements, participants were categorized into three groups: normal (*n* ═ 79), osteopenia (*n* ═ 93), and OP (*n* ═ 115). A BMD T-score of ≥ −1.0 was considered normal, a score between −2.5 and −1.0 indicated osteopenia, and a score of ≤ −2.5 defined OP [22].

### Inclusion and exclusion criteria

Inclusion criteria were as below: 1) diagnosed as PM women; 2) meeting OP-associated diagnostic criteria [23] and diagnosed with OP for the first time [based on the results of BMD measurement by dual-energy X-ray absorptiometry (DXA), where the T-value/Z-value of the BMD determined by DXA was ≤ −2.5]; 3) complete clinical data.

Exclusion criteria were as follows: 1) comorbidities of other chronic disorders or endocrine disorders influencing bone metabolism, comprising malignant tumors, hyperthyroidism and diabetes mellitus; 2) receiving relevant treatments affecting bone turnover within 3 months; 3) administration of medications influencing bone metabolism, such as calcium-containing medications, bisphosphonates, androgen, estrogen, vitamin D and glucocorticosteroids; 4) dysfunctions of important organs such as the kidney and liver.

### Clinical data collection

The basic characteristics of the enrolled subjects were collected, including age, serum calcium levels, body mass index (BMI), years since menopause (YSM), serum phosphoruslevels, and BMD of the femoral neck and lumbar spine, assessed using DXA (Discovery W, Hologic, Waltham, MA, USA). Menopause was defined as the permanent cessation of menstruation (unrelated to pregnancy) due to ovarian failure. Menopausal status was determined based on self-report, medical records, or both, and defined as the absence of menstruation for at least 12 consecutive months in women over 40 years of age. A 6 mL fasting venous blood sample was collected from the antecubital vein of each eligible subject upon admission and divided equally into two parts. One portion was placed into collection tubes without anticoagulant and left to stand at room temperature for 30 min to allow clotting. It was then centrifuged at 2000 r/min for 20 min, and the resulting serum was stored at −80^∘^C. The other portion was placed into tubes containing anticoagulant, gently mixed, and left to stand for 1 hour. Following centrifugation, the supernatant (plasma) was collected and stored at −80^∘^C.

### Enzyme-linked immunosorbent assay (ELISA)

Serum levels of procollagen type I N-propeptide (PINP) and β-C-terminal telopeptide of type I collagen (β-CTX) were measured using ELISA with an FK-SY96S multifunctional ELISA analyzer (Fangke Instruments Co., Ltd., Weifang, Shandong, China). The ELISA kits were provided by Mlbio (Shanghai, China). Plasma levels of Trp, 5-HIAA, 5-HT, and 5-HTP were determined using corresponding ELISA kits from Camilo (Nanjing, Jiangsu, China) and Jianglaibio (Shanghai, China), following the manufacturers’ instructions.

### Ethical statement

This study was approved by the Ethics Committee of The Tenth Affiliated Hospital, Southern Medical University (Dongguan People’s Hospital) and conducted in accordance with the principles of the Declaration of Helsinki. It involved the use of existing data from medical records, for which informed consent had been exempted during the original data collection. In line with ethical guidelines and the approval of the Institutional Review Board (IRB) at The Tenth Affiliated Hospital, Southern Medical University (Dongguan People’s Hospital), informed consent for the current study was waived.

### Statistical analysis

Statistical analyses and data visualization were performed using SPSS version 27.0 (IBM Corp., Armonk, NY, USA) and GraphPad Prism version 9.5 (GraphPad Software, San Diego, CA, USA). Measurement data with normal distribution, as verified by the Kolmogorov–Smirnov test, were expressed as mean ± standard deviation. One-way ANOVA followed by Tukey’s post hoc test was used for multiple group comparisons. Pearson correlation analysis was conducted to assess the relationships between 5-HTP, Trp, 5-HIAA, and 5-HT levels and PINP, β-CTX, femoral neck BMD, and lumbar spine BMD in PMOP patients. A multivariate logistic regression model was used to identify independent risk factors for PMOP. Receiver operating characteristic (ROC) curves were generated to evaluate the predictive value of 5-HTP, 5-HT, and 5-HIAA, individually and in combination, for PMOP. Diagnostic accuracy was assessed using MedCalc statistical software (version 20.0.15; MedCalc Software Ltd., Ostend, Belgium), and differences in the areas under the ROC curves (AUCs) were compared using the DeLong test. Kaplan–Meier curves were employed to analyze the impact of expression differences in 5-HT precursors and metabolites on the risk of PMOP. A two-tailed *P* value < 0.05 was considered statistically significant.

## Results

### Comparisons of the baseline characteristics of patients in the normal, OP and osteopenia groups

The enrolled PM women were grouped into the normal, OP and osteopenia groups based on the results of BMD measurements. In terms of BMI, age, and serum calcium and serum phosphorus levels, the three groups represented no statistically noticeable variations (all *P* > 0.05); by contrast, YSM, and PINP and β-CTX levels were higher while femoral neck BMD, and lumbar spine BMD were lower in the OP group than in the normal and osteopenia groups (all *P* < 0.01) (Table 1).

**Table 1:** Comparisons of baseline characteristics of patients in the normal, osteopenia and OP groups

**Clinical data**	**Normal group (*n* ═ 79)**	**Osteopenia group (*n* ═ 93)**	**OP group (*n* ═ 115)**	***P***
Age (years)	61.80 ± 4.38	62.09 ± 5.09	62.43 ± 6.04	0.715
BMI (kg/m^2^)	24.09 ± 2.53	23.68 ± 2.47	23.54 ± 2.96	0.372
YSM (years)	10.58 ± 3.07	11.00 (2.00, 23.00)	14.09 ± 3.56^aabb^	< 0.001
Lumbar spine BMD (g/cm^2^)	1.03 ± 0.15	0.87 ± 0.14^aa^	0.80 ± 0.10^aabb^	< 0.001
Femoral neck BMD (g/cm^2^)	0.95 ± 0.11	0.86 ± 0.11^aa^	0.78 ± 0.09^aabb^	< 0.001
Serum phosphorus (mmol/L)	1.05 ± 0.12	1.04 ± 0.14	1.02 ± 0.11	0.262
Serum calcium (mmol/L)	2.35 ± 0.16	2.33 ± 0.11	2.32 ± 0.14	0.329
β-CTX (ng/mL)	0.34 ± 0.11	0.37 (0.06, 0.59)	0.46 ± 0.13^aabb^	< 0.001
PINP (ng/mL)	33.25 ± 5.36	36.89 ± 6.45^aa^	47.51 ± 8.05^aabb^	< 0.001

Measurement data conforming to a normal distribution were depicted as mean ± standard deviation. One-way ANOVA was employed for analyzing inter-group comparisons, followed by Tukey’s multiple comparison test. Measurement data that did not display normal distribution were presented as median (minimum value-maximum value) and compared by the Kruskal–Wallis with Dunn’s multiple comparison test. ^a^*P* < 0.05, ^aa^*P* < 0.01, vs the normal group. ^b^*P* < 0.05, ^bb^*P* < 0.01, vs the osteopenia group. BMI: Body mass index; BMD: Bone mineral density; YSM: Years since menopause; β-CTX: β-C-terminal telopeptide of type 1 collagen; PINP: Procollagen type I N-propeptide.

**Figure 1.**
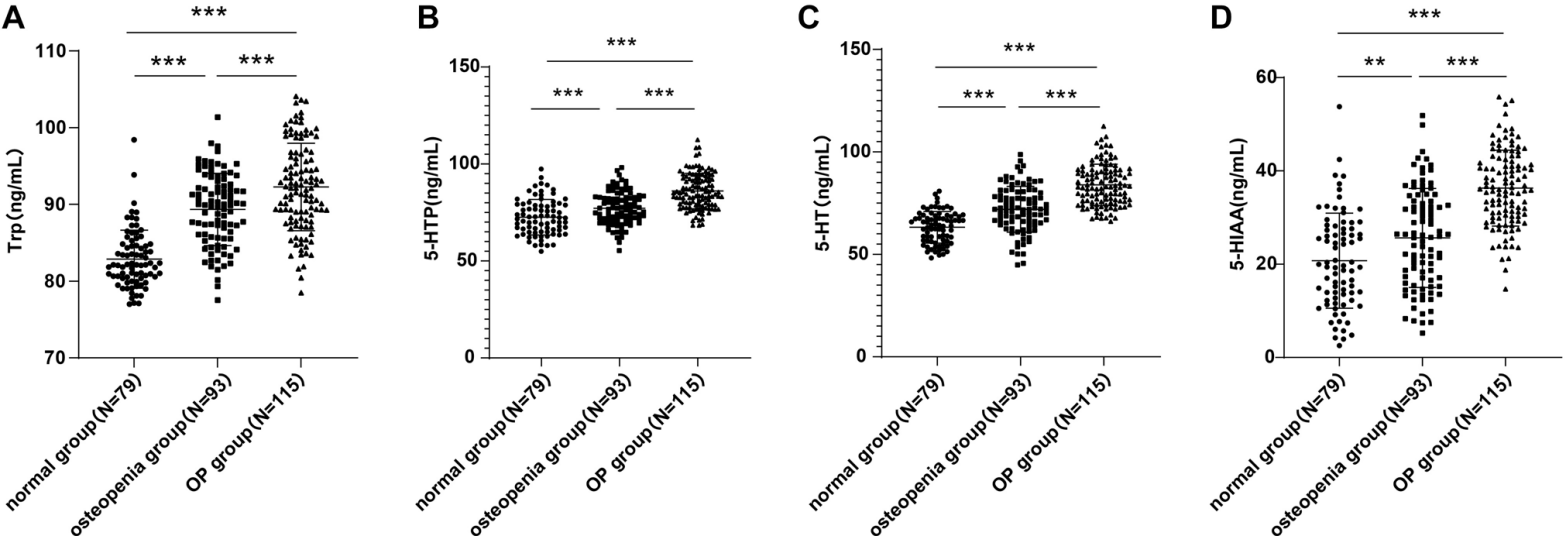
**Plasma levels of 5-HT, Trp, 5-HTP and 5-HIAA in PM women among the normal, osteopenia and OP groups.** Levels of Trp, 5-HT, 5-HTP and 5-HIAA were assessed by ELISA and the differences in plasma levels of Trp (A), 5-HTP (B), 5-HT (C) and 5-HIAA (D) were compared among the normal, osteopenia and OP groups. Data were presented as mean ± standard deviation, and compared by one-way ANOVA with Tukey’s post hoc test. ***P < 0.001. 5-HTP: 5-hydroxytryptophane; 5-HT: 5-hydroxytryptamineserotonin; 5-HIAA: 5-hydroxyindoleacetic acid; OP: Osteoporosis; ELISA: Enzyme-linked immunosorbent assay.

### Trp, 5-HT, 5-HTP and 5-HIAA levels were augmented in the plasma of PMOP patients, and prominently correlated with bone turnover markers (****BTMs)

We then compared the plasma levels of 5-HTP, Trp, 5-HT and 5-HIAA among the three groups and unveiled enhancements in the levels of these indicators in the OP group relative to the osteopenia and normal groups, with the former showing higher levels than the latter (all *P* < 0.05, Figure 1A–1D). Further, Pearson’s correlation analysis implied that plasma 5-HT, Trp, 5-HTP and 5-HIAA had positive correlations with PINP and β-CTX in patients with PMOP, and negative correlations with femoral neck BMD and lumbar spine BMD (all *P* < 0.05, Table 2).

**Table 2:** Analyses on the correlations of plasma 5-HT precursors and metabolites with BTMs and BMD in patients with PMOP

**Indicators**	**Trp**		**5-HTP**		**5-HT**		**5-HIAA**	
	***r* value**	***P* value**	***r* value**	***P* value**	***r* value**	***P* value**	***r* value**	***P* value**
Lumbar spine BMD	−0.418	<0.001	−0.429	<0.001	−0.533	<0.001	−0.477	<0.001
Femoral neck BMD	−0.208	0.026	−0.422	<0.001	−0.323	<0.001	−0.398	<0.001
β-CTX	0.477	<0.001	0.343	<0.001	0.565	<0.001	0.422	<0.001
PINP	0.499	<0.001	0.562	<0.001	0.766	<0.001	0.631	<0.001

5-HT: 5-hydroxytryptamine; BMD: Bone mineral density; PINP: Procollagen type I N-propeptide; β-CTX: β-C-terminal telopeptide of type 1 collagen; Trp: Tryptophan; 5-HTP: 5-hydroxytryptophane; 5-HIAA: 5-hydroxyindoleacetic acid; PMOP: Postmenopausal osteoporosis.

### Elevated levels of 5-HT precursors and metabolites were independent risk factors for the occurrence of OP** **in PM women

To precisely assess the impact of 5-HT precursors and metabolites on the occurrence of OP in PM women, we conducted multivariate logistic regression analyses. The presence of PMOP (0 ═ no, 1 ═ yes) was used as the dependent variable, while YSM, 5-HIAA,5-HTP, Trp, 5-HT, PINP, β-CTX, femoral neck BMD, and lumbar spine BMD were included as independent variables. The results indicated that increased YSM and elevated levels of β-CTX, PINP, 5-HIAA, 5-HTP, and 5-HT were independent risk factors for PMOP. In contrast, higher lumbar spine and femoral neck BMD values were identified as protective factors (Table 3).

**Table 3:** Multivariate logistic regression analyses on the OP occurrence in PM women

**Independent variable**	***P* value**	**OR value**	**95% CI**
YSM	<0.001	1.426	1.242∼1.637
Trp	0.795	1.012	0.926∼1.105
5-HTP	0.046	1.057	1.001∼1.116
5-HT	0.039	1.046	1.002∼1.092
5-HIAA	0.029	1.049	1.005∼1.095
β-CTX	0.045	25.441	1.074∼602.398
PINP	0.001	1.132	1.049∼1.222
Lumbar spine BMD	0.043	0.026	0.001∼0.894
Femoral neck BMD	0.041	0.014	0.000∼0.839

YSM: Years since menopause; BMD: Bone mineral density; PINP: Procollagen type I N-propeptide; β-CTX: β-C-terminal telopeptide of type 1 collagen; Trp: Tryptophan; 5-HTP: 5-hydroxytryptophane; 5-HT: 5-hydroxytryptamineserotonin; 5-HIAA: 5-hydroxyindoleacetic acid; OP: Osteoporosis.

### 5-HT precursors and metabolites provided diagnostic value for PMOP

**Figure 2.**
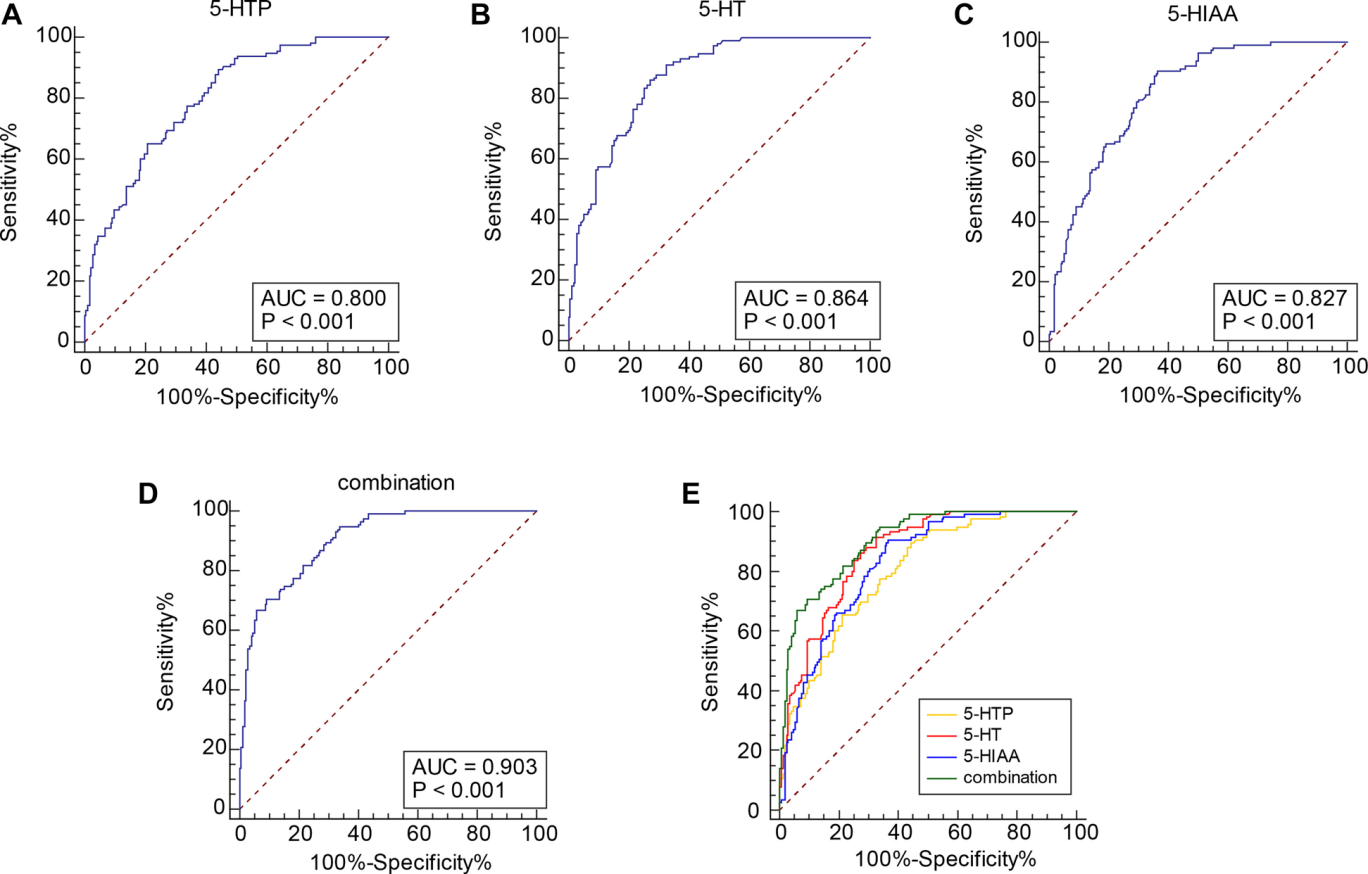
**Predictive values of 5-HT precursors and metabolites alone or in combination for PMOP occurrence.** ROC curves were plotted for analyzing the predictive value of plasma 5-HTP (A), 5-HT (B), 5-HIAA (C) levels, and the combined assay (D) for PMOP. The AUCs of these indicators (E) were compared. 5-HTP: 5-hydroxytryptophane; 5-HT: 5-hydroxytryptamineserotonin; 5-HIAA: 5-hydroxyindoleacetic acid; PMOP: Postmenopausal osteoporosis; ROC: Receiver operating characteristic.

Subsequently, we further investigated the predictive value of 5-HT precursors and metabolites in diagnosing PMOP. ROC curve analysis showed that the AUCs for 5-HTP, 5-HT, and 5-HIAA were 0.800 (95% CI: 0.749–0.845), 0.864 (95% CI: 0.819–0.902), and 0.827 (95% CI: 0.778–0.869), respectively. Their corresponding cut-off values were 76.02, 73.03, and 26.68, with sensitivities of 89.57%, 86.09%, and 90.43%, and specificities of 55.81%, 72.67%, and 63.37% (Table 4, Figure 2A–2C). Notably, the combined analysis of 5-HT precursors and metabolites yielded a higher AUC than any individual marker (all *P* < 0.05; Table 4, Figure 2D–2E). These findings indicate that 5-HT precursors and metabolites possess strong diagnostic value for PMOP, and that their combined use enhances diagnostic accuracy compared to using either group alone.

**Table 4:** Comparative analyses on AUCs of the 5-HT precursors and metabolites alone and joint detection for predicting PMOP

**Items**	**AUC**	**95% CI**	**Sensitivity**	**Specificity**
5-HTP	0.800	0.749∼0.845	89.57	55.81
5-HT	0.864	0.819∼0.902	86.09	72.67
5-HIAA	0.827	0.778∼0.869	90.43	63.37
Combination	0.903	0.862∼0.934	66.96	94.19
5-HTP vs combination	*P* < 0.001			
5-HT vs combination	*P* < 0.001			
5-HIAA vs combination	*P* < 0.001			

The differences of AUC of multiple ROC curves were compared using the DeLong test in MedCalc 20.0.15 software (MedCalc software, Ostend, Belgium). Combination represented the combined analysis of of 5-HTP, 5-HT, and 5-HIAA. 5-HTP: 5-hydroxytryptophane; 5-HIAA: 5-hydroxyindoleacetic acid; PMOP: Postmenopausal osteoporosis.

### High levels of 5-HT precursors and metabolites increased the risk of PMOP

**Figure 3.**
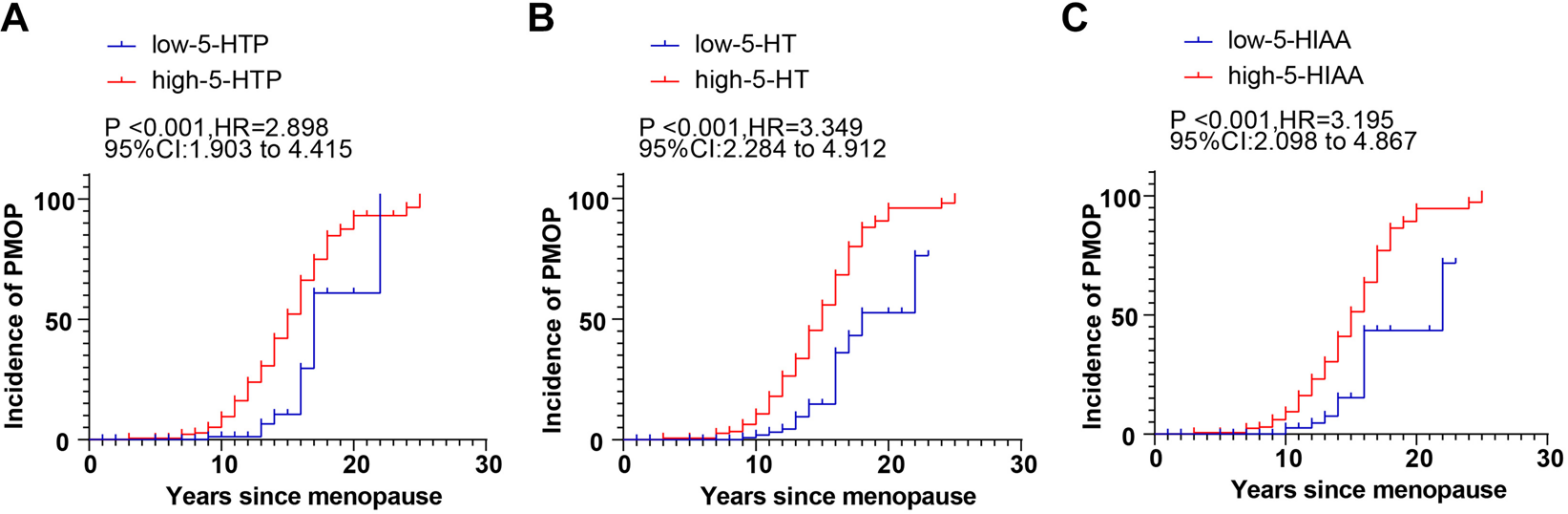
**Influence of plasma levels of 5-HT precursors and metabolites on the occurrence of PMOP analyzed by Kapan–Meier curves.** The influence of plasma 5-HTP (A), 5-HT (B) and 5-HIAA (C) levels on the risk of PMOP occurrence analyzed by Kapan–Meier curves. 5-HTP: 5-hydroxytryptophane; 5-HT: 5-hydroxytryptamineserotonin; 5-HIAA: 5-hydroxyindoleacetic acid; PMOP: Postmenopausal osteoporosis.

Finally, we categorized patients into two groups based on the median value of YSM: the YSM ≥ 12 years group (*n* ═ 168) and the YSM < 12 years group (*n* ═ 119). We then compared their plasma levels of 5-HTP, 5-HT, and 5-HIAA. The results showed significantly higher plasma levels of 5-HTP, 5-HT, and 5-HIAA in the YSM ≥ 12 years group compared to the YSM < 12 years group (all *P* < 0.05, Table 5). In addition, patients were divided into high and low expression groups based on the ROC-derived cut-off values for 5-HT precursors and metabolites (76.02 ng/mL for 5-HTP, 73.03 ng/mL for 5-HT, and 26.68 ng/mL for 5-HIAA). Kaplan–Meier curves were used to analyze the risk of PMOP occurrence in patients with different expression patterns of these biomarkers. Patients with plasma 5-HTP ≥ 76.02 ng/mL, 5-HT ≥ 73.03 ng/mL, or 5-HIAA ≥ 26.68 ng/mL exhibited survival curves that shifted to the left compared to those with lower levels (all *P* < 0.001, Figure 3A–3C). These findings suggest that levels of 5-HTP, 5-HT, and 5-HIAA increase with YSM, and their elevated levels (5-HTP: HR ═ 2.898; 5-HT: HR ═ 3.349; 5-HIAA: HR ═ 3.195) significantly shorten the time to PMOP occurrence, thereby increasing PMOP risk. Overall, these results indicate that targeting these biomarkers may offer a potential strategy for delaying the onset of PMOP.

**Table 5:** Comparison of 5-HTP, 5-HT, and 5-HIAA levels in the YSM ≥12 years group and the YSM <12 years group

**Item**	**YSM ≥ 12 years group (*n* ═ 168)**	**YSM < 12 years group (*n* ═ 119)**	***P* value**
5-HTP (ng/mL)	81.02 ± 10.08	77.30 ± 10.93	0.0031
5-HT (ng/mL)	75.74 ± 13.12	72.41 ± 12.65	0.0325
5-HIAA (ng/mL)	31.48 ± 10.30	21.79 (2.56, 52.23)	<0.0001

Measurement data conforming to normal distribution were expressed as mean ± standard deviation. Comparison between two groups was performed using unpaired *t*-test. Measurement data that do not display normal distribution are presented as median (minimum value-maximum value) and compared by Mann–Whitney test. YSM: Years since menopause; 5-HTP: 5-hydroxytryptophane; 5-HT: 5-hydroxytryptamineserotonin; 5-HIAA: 5-hydroxyindoleacetic acid.

## Discussion

PMOP imposes significant financial and physical burdens on aging women, with approximately one-third of women over 50 years of age experiencing osteoporotic fractures [24]. Emerging evidence suggests that 5-HT plays a role in the onset and progression of OP by modulating bone mass [25, 26]. In this study, we demonstrated that changes in plasma levels of 5-HT precursors and metabolites can aid in the diagnosis of OP. BMD—defined as the amount of mineral per unit of bone—is a key determinant of bone strength and a primary indicator of osteopenia or OP [27]. Patients with OP typically exhibit reduced BMD and bone strength, which increases fracture risk from low-energy injuries [28]. Additionally, biochemical BTMs, including β-CTX and PINP, are elevated in women with OP-related fractures [29]. Specifically, serum β-CTX is a recognized marker of bone resorption, while PINP and BALP are associated with bone formation and osteoblast activity [30, 31]. OP patients often present with elevated PINP and β-CTX levels, indicating high bone turnover [32]. In our study, PMOP patients exhibited significantly higher levels of β-CTX, PINP, and YSM, along with lower femoral neck and lumbar spine BMD, compared to individuals with normal bone mass or osteopenia. However, no statistically significant differences were observed among the groups in terms of BMI, age, serum calcium, or serum phosphorus levels. PMOP is characterized by reduced bone mass and mineral density, largely driven by estrogen deficiency, as well as genetic factors, smoking, diet, exercise, alcohol use, advanced age, weight loss, impaired calcium absorption [2, 3], and immune alterations [4]. Importantly, BMD is not directly associated with BMI, age, or serum calcium and phosphorus levels [33]. Rather, changes in bone mass in postmenopausal women are primarily influenced by estrogen levels, osteogenic differentiation, osteoclast activity, and related factors [2, 34, 35]. The lack of significant differences in BMI, age, and serum calcium and phosphorus among the groups may reflect the fact that these variables are not direct determinants of osteopenia or OP. Moreover, our retrospective, single-center study with a relatively small sample size may have influenced the statistical power of our findings. Notably, we observed significantly elevated plasma levels of 5-HTP, Trp, 5-HIAA, and 5-HT in PMOP patients compared to those with normal bone mass or osteopenia. To our knowledge, this is the first study to report significant alterations in 5-HT precursors and metabolites in PMOP. BTMs are sensitive indicators of bone formation and resorption, and their elevation is commonly linked to increased fracture risk [36, 37]. 5-HT is recognized as a key regulator of bone turnover [38]. Previous studies have reported that elevated 5-HT, 5-HIAA, and 5-HTP levels in PMOP patients are associated with increased PINP and β-CTX levels. These metabolites show a negative correlation with BMD and a positive correlation with BTMs [39]. Furthermore, selective inhibition of 5-HT has been shown to reduce BTMs and fracture risk [39–44]. These findings align with our results, showing positive associations of 5-HT, 5-HIAA, and 5-HTP with β-CTX and PINP, and negative associations with lumbar spine and femoral neck BMD in PMOP patients.

Our multivariate regression analysis revealed that increased YSM, elevated levels of 5-HT precursors and metabolites, as well as higher β-CTX and PINP levels, were independent risk factors for PMOP. In contrast, higher BMD at the lumbar spine and femoral neck served as protective factors. Notably, elevated plasma levels of 5-HT precursors and metabolites were associated with a shorter YSM before PMOP onset and an increased risk of OP in postmenopausal women. Previous studies have reported that postmenopausal subjects with OP, osteopenia, or normal bone mass exhibit different YSM durations [45]. YSM has also been closely linked to the risk of osteoporotic fractures in postmenopausal women [46]. Furthermore, low total hip and lumbar spine BMD have been identified as risk factors for both primary and recurrent fractures in women with PMOP [47]. In both men and women with OP, serum levels of PINP and β-CTX have been shown to be independent risk factors [48]. However, few studies have investigated the predictive value of 5-HT precursors and metabolites in diagnosing PMOP. To our knowledge, this is the first study to identify 5-HT precursors and metabolites as independent risk factors associated with PMOP occurrence. In future research, these markers—especially when used in combination—may aid in more accurately assessing PMOP risk.

## Conclusion

This study found that 5-HT precursors and metabolites were significantly correlated with bone turnover markers and BMD. They were identified as independent risk factors for the development of PMOP. Elevated plasma levels of 5-HT precursors and metabolites were strongly associated with a shorter YSM period before the onset of PMOP and an increased risk of OP in postmenopausal women. These findings suggest the clinical potential of 5-HT precursors and metabolites as plasma biomarkers for the diagnosis and treatment of PMOP. Additionally, the study offers potential targets for delaying the onset of OP in postmenopausal women, particularly in relation to YSM. However, the study has several limitations. First, it was a retrospective, single-center study with a relatively small sample size. Therefore, future research should involve multiple centers and larger cohorts. Second, although YSM is a possible influencing factor for PMOP, we were unable to investigate the impact of menopause duration due to limited time and work constraints. Lastly, the cellular and molecular mechanisms of 5-HT precursors and metabolites were not explored in this study because of time and funding limitations, but they will be the focus of future research.

## Supplemental data

**Figure S1.**
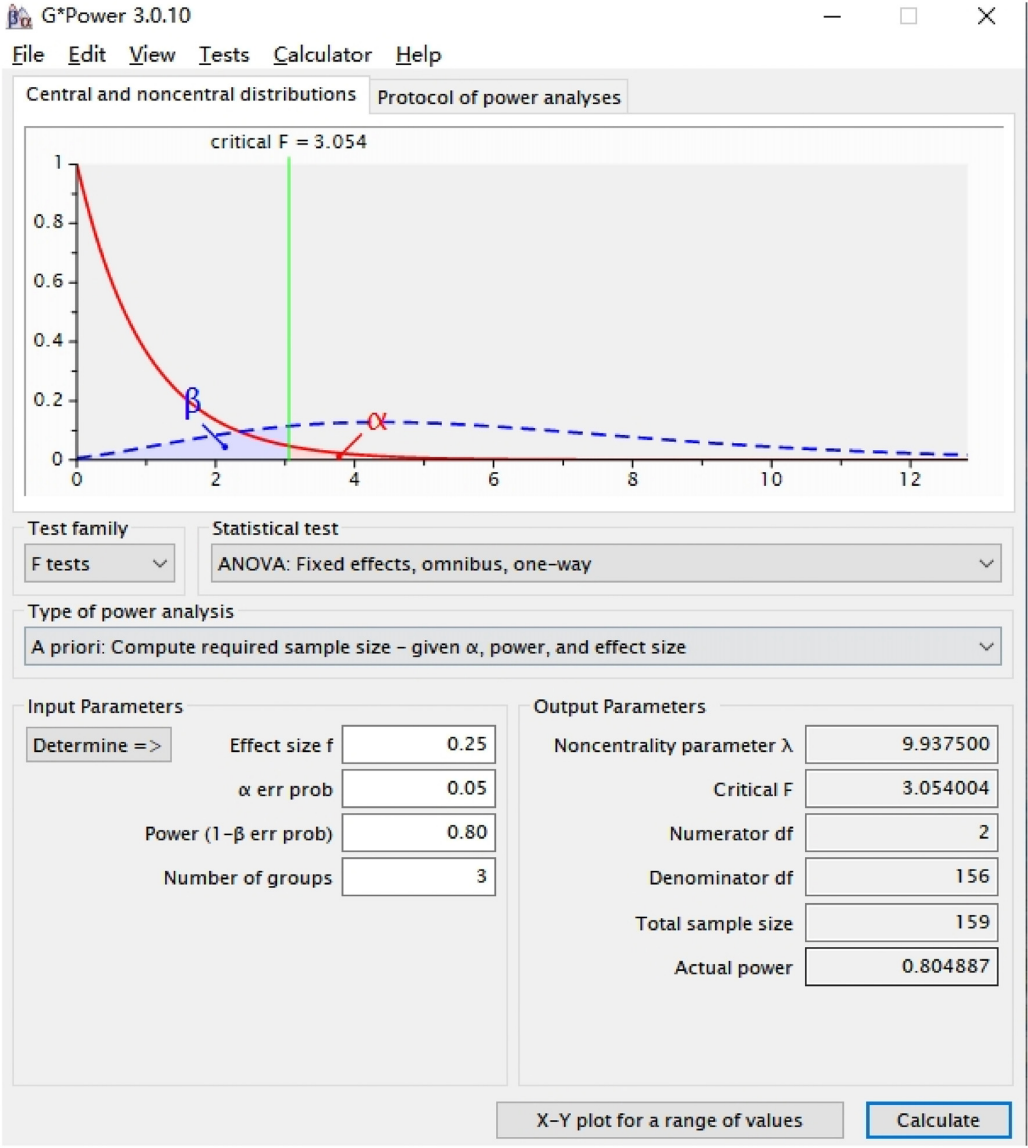
Sample size estimation by G*Power.

## Data Availability

The data supporting the findings of this study are available from the corresponding author upon reasonable request.
